# *Candida* Chorioamnionitis Leads to Preterm Birth and Adverse Fetal-Neonatal Outcome

**DOI:** 10.1155/2017/9060138

**Published:** 2017-10-17

**Authors:** Yohei Maki, Midori Fujisaki, Yuichiro Sato, Hiroshi Sameshima

**Affiliations:** ^1^Department of Obstetrics and Gynecology, Faculty of Medicine, University of Miyazaki, Miyazaki, Japan; ^2^Department of Diagnostic Pathology, Faculty of Medicine, University of Miyazaki, Miyazaki, Japan

## Abstract

*Candida* chorioamnionitis is rare but can lead to neonatal infection, high mortality, and neurodevelopmental impairment. We aimed to investigate maternal clinical features and perinatal outcomes and discuss future management strategies. We reviewed the medical records of women with* Candida* chorioamnionitis at our hospital over a 10-year period (*n* = 9) and previous published case reports and case series. The most prevalent* Candida* species was* C. albicans* (71.3% of the all cases). The most prevalent predisposing condition was preterm premature rupture of membranes (31/123, 25.2%), followed by pregnancy with a retained intrauterine contraceptive device (26/123, 21.1%) and pregnancy after in vitro fertilization (25/123, 20.3%). Preterm labor was the most common symptom (52/123, 42.3%), and only 13% of cases involved fever. Of the infants, 27% of the singletons and 23.8% of the twins were born before 22 gestational weeks, while 60% of the singletons and 76.2% of the twins were born at 22–36 weeks. The median birth weight of the babies born after 22 weeks was 1230 g. The mortality rates of the singletons and twins born after 22 weeks of gestation in the year 2000 or later were 28.6% and 52.4%, respectively. Antenatal treatment for* Candida* chorioamnionitis has not been established.

## 1. Introduction

Despite the high incidence of vulvovaginal candidiasis during pregnancy (13–20%) [1, 2],* Candida* species rarely cause chorioamnionitis. A recent neonatal study demonstrated that chorioamnionitis is an important risk factor of invasive early-onset candidiasis in extremely low-birth-weight infants, which leads to high mortality (71%) and neurodevelopmental impairment rates (86%) [3]; thus, the establishment of antenatal management is crucial. Since the first case of chorioamnionitis caused by* Candida albicans* that resulted in preterm birth and neonatal death was reported in 1958 [4], many case reports and short case series have been published. However, owing to the rarity of the disease, only two studies involving small groups of patients, a case series of 32 patients [5] and a case control study of 18 patients [6], have been published to date. Therefore, the clinical features of* Candida* chorioamnionitis are not well understood and management of the disease has not been established yet. Here, we present a case series of* Candida* chorioamnionitis in the past 10 years at our hospital and review the previously published case reports and case series. This study aimed to investigate the maternal clinical features and perinatal outcomes of these cases and discuss future management strategies.

## 2. Materials and Methods

We reviewed the medical records of women with* Candida* chorioamnionitis who attended the University of Miyazaki Hospital, a tertiary medical center in Miyazaki, Japan, between 2007 and 2016. The ethics committee of the Faculty of Medicine of University of Miyazaki approved this study (registration number O-0135).* Candida* chorioamnionitis is diagnosed on the basis of one or more of the following criteria: (1)* Candida* species are isolated from amniotic fluid obtained using transabdominal amniocentesis; (2) clinical or histological chorioamnionitis along with fetal/neonatal/placental culture test results positive for* Candida* species; or (3) histological chorioamnionitis (defined by polymorphonuclear leukocytes in the chorion or chorioamnion) and/or funisitis (defined by polymorphonuclear leukocytes in the wall of a blood vessel in the umbilical cord or the chorionic plate) involving yeast forms with pseudohyphae on periodic acid-Schiff staining, which is characteristic of* Candida* species. Cases of neonatal congenital candidiasis without any signs of maternal clinical/histological chorioamnionitis were excluded owing to the possibility of a birth canal infection during delivery rather than an in utero infection. In our hospital, we routinely perform transabdominal amniocentesis in women with preterm labor with or without preterm premature rupture of membranes (pPROM) to exclude intra-amniotic infection after obtaining their informed consent, unless other comorbid conditions such as placental position, inadequate amniotic space, or large bag protrusion into the vagina are present since we have reported that management with amniocentesis for women with preterm labor with intact membranes might improve neonatal outcome born between 22 and 28 weeks of gestation [7]. Umbilical cord blood cultures were immediately collected after delivery from all the women with preterm delivery and from those with term delivery who had signs of clinical chorioamnionitis, such as fever, leukocytosis, and maternal/fetal tachycardia. Histopathological examinations of the placentas/umbilical cords from these women were also performed. Periodic acid-Schiff staining was additionally performed after hematoxylin and eosin staining when a* Candida* infection was suspected.

We searched Medline, PubMed, and Google Scholar for case reports and case series in the English literature that were published till December 2016, using the terms “chorioamnionitis,” “intra-amniotic infection,” “*Candida* species,”* “Candida albicans,” “Candida glabrata,” “Torulopsis glabrata,”* and “congenital* candidiasis*”. We also searched the references of the published case series. We used the above-mentioned criteria for* Candida* chorioamnionitis and excluded duplicate cases and reports without adequate information about the maternal clinical course.

## 3. Results

### 3.1. Case Series

Between January 2007 and December 2016, 2717 deliveries were performed in our hospital. During this period, we had nine cases (0.3%) of* Candida* chorioamnionitis (Table 1). The isolated organisms were* C. albicans* in six women,* C. glabrata* in two women, and* C. famata* in one woman. Eight cases were antenatally diagnosed using transabdominal amniocentesis. One case, in which a transabdominal amniocentesis was not conducted, was diagnosed as histological chorioamnionitis and funisitis with* Candida* infection using an umbilical blood culture. This case involved dichorionic diamniotic (Di-Di) twins, of whom only the first infant had* C. albicans* infection.

#### 3.1.1. Maternal Clinical Features

The mean maternal age was 32 years (range, 26–38 years). Three cases of pregnancy after in vitro fertilization (IVF) embryo transfer and one case of pPROM occurred. One woman had pregestational diabetes, and another had gestational diabetes; both conditions were well controlled. None of the patients had an intrauterine contraceptive device (IUCD) or a cervical cerclage. Eight patients were hospitalized for preterm labor. None of the patients were febrile. In three patients, the white blood cell counts reached >15,000/mm^2^ and the C-reactive protein (CRP) levels ranged from 0.51 to 7.51 mg/dL (mean, 3.1 mg/dL). One patient had an induced abortion at 21 weeks of gestation. In seven women diagnosed using transabdominal amniocentesis, delivery was immediately induced by oxytocin or performed via cesarean section after diagnosis. The woman with Di-Di twins who did not undergo transabdominal amniocentesis had a spontaneous delivery. No women received antenatal corticosteroids.

#### 3.1.2. Fetal/Neonatal Outcome

The case of induced abortion was excluded from the analysis. The mean gestational age at delivery was 24.5 weeks (range, 22–33 weeks). The mean birth weight was 690 g (range, 498–1912 g). One of the twins, neither of whom had been diagnosed prenatally, had a positive umbilical blood culture, and the other three infants had cutaneous candidiasis. All the infants received intravenous fluconazole or micafungin and did not test positive in the following blood and surface cultures. Two infants died at the neonatal intensive care unit, one of liver hemorrhage and the other of intestinal perforation. Two infants were at risk of severe neurological impairment at the time of discharge.

### 3.2. Literature Review

We identified 123 cases (102 singletons [4, 8–76]/21 twins [49, 62, 69, 77–90]) with* Candida* chorioamnionitis, including our nine cases for whom adequate maternal clinical data were available.

#### 3.2.1. Microbiological Characteristics

Cultural identification was conducted in 101 cases, while the remaining 22 cases were diagnosed using histopathological placenta/umbilical findings without cultural identification. The most prevalent species was* C. albicans* (71.3% [72/101]), followed by* C. glabrata* (21.7% [22/101]),* C. tropicalis* (3% [3/101]),* C. lusitaniae* (2% [2/101]),* C. parapsilosis* (2% [2/101]),* C. famata* (1% [1/101]), and* C. kefyr* (1% [1/101]; Table 2). Coinfection with* C. albicans* and* C. parapsilosis* was found in two cases [56, 83], one of which was a twin pregnancy.* C. albicans* was identified in one infant, while* C. parapsilosis* was identified in another [83].

#### 3.2.2. Maternal Clinical Features

The maternal clinical features are shown in Table 3. The median maternal age was 29 years (range, 16–47 years). Five patients had pregestational or gestational diabetes, and one patient received prednisolone for suspected immunological miscarriages. None of the patients had human immunodeficiency virus infection or other immunosuppressive diseases. The most prevalent predisposing condition was pPROM in 25.2% (31/123) of the patients, followed by pregnancy with a retained IUCD (21.1% [26/123]), pregnancy after IVF (20.3% [25/123]), a history of transabdominal amniocentesis during the current pregnancy (8.9% [11/123]), or cervical cerclage (6.5% [8/123]). Of the cases, 11.4% (14/123) had a history of treatment for vulvovaginal candidiasis during the current pregnancy. Preterm labor was the most common symptom (42.3% [52/123]), while only 13% (16/123) of the cases involved fever. Cervical dilatation without uterine contraction was found in 8.9% (11/123) of the cases, while 9.8% (12/123) had no symptoms. Laboratory data were unavailable in most of the reports. No cases of maternal death occurred.

#### 3.2.3. Fetal/Neonatal Outcome

The distribution of the gestational ages at delivery is shown in Table 4. Two singletons were excluded because of missing information on gestational age. Birth before 22 weeks occurred in 27% (27/100) of the singletons and 23.8% (5/21) of the twins, while birth between 22 and 36 weeks occurred in 60% (60/100) of the singletons and 76.2% (16/21) of the twins. The distributions of the singletons and twin births were as follows: between 22 and 23 weeks, 11% (11/100) and 9.5% (2/21), respectively; between 24 and 27 weeks, 24% (24/100) and 19% (4/21), respectively. The median birth weight of the babies born after 22 weeks was 1,230 g (range, 425–4350 g).

**Figure 1:**
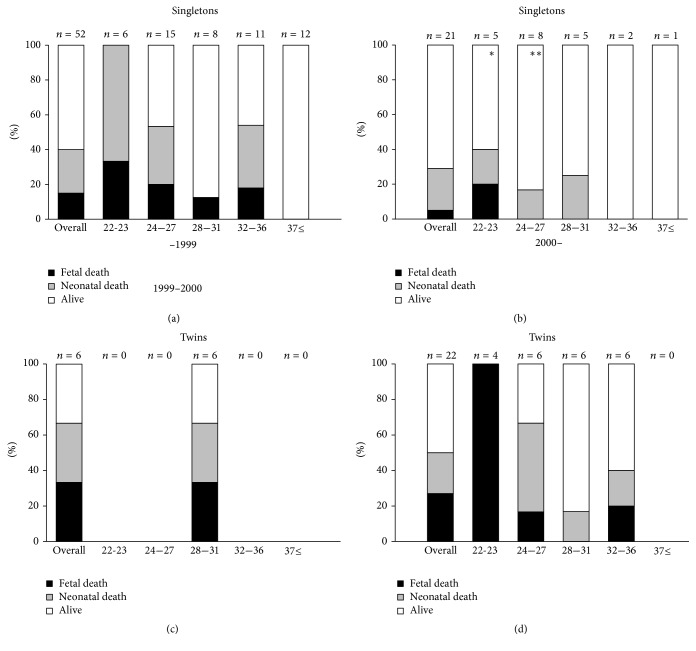
Perinatal mortality rate of infants with* Candida* chorioamnionitis born after 22 weeks' gestation in the literature review. (a) Singletons born before 2000; (b) singletons born in 2000 or later; (c) twins born before 2000; (d) twins born in 2000 or later. Black bar: fetal death; gray bar: neonatal death; white bar: alive for the first 28 days of life. ^*∗*^One infant died on day 59. ^*∗∗*^One infant died on day 42. One twin of five twins without chorioamnionitis was excluded from analysis.

The mortality rates of the singletons and twins born after 22 weeks are shown in Figure 1. Since the description of the clinical features of the infants varied among the reports, we were unable to obtain sufficient data to evaluate the prevalence of congenital candidiasis caused by* Candida* chorioamnionitis. Therefore, we analyzed all infants associated with* Candida* chorioamnionitis. The mortality rate of the singletons born after 22 weeks since 2000 was 29%, while that of the twins was 50%. In five cases of twins, only one twin was associated with* Candida* chorioamnionitis, and another twin had no signs of chorioamnionitis or funisitis. These five infants without chorioamnionitis or funisitis were excluded from the analysis. The overall mortality rate of the singletons since the year 2000 improved to 28.6% from 40.4% before the year 2000. The overall mortality rate of the twins since 2000 was 52.4%, higher than that of the singletons.

#### 3.2.4. Antenatal Treatment

Antenatal treatment was conducted in 13 cases, including four of the twins (Table 5) [41, 44, 64, 71, 73–76, 81, 87–89]. All the cases were diagnosed using amniotic fluid culture obtained through transabdominal amniocentesis at a median gestational age of 22 weeks (range, 19–26 weeks). Fluconazole was the most prevalent agent used in six cases, followed by amphotericin B in five cases and ketoconazole and micafungin in one case each. The administration routes included oral, intravenous, vaginal, transabdominal intra-amniotic, and transcervical intra-amniotic. The prolongation period varied from 0 days to 8 weeks. Six of 13 cases with antenatal treatment succeeded in having alive infants.

## 4. Discussion

### 4.1. Epidemiology

The prevalence of* Candida* chorioamnionitis at our institution was 0.3% of all pregnant women. This finding is consistent with the 0.5% prevalence reported in a previous study [6]. However, considering that both institutions are regional referral centers, we suspect that the true prevalence is less frequent than 0.3–0.5%.

### 4.2. Maternal Clinical Features

The presence of an IUCD during pregnancy, an established risk factor of intra-amniotic* Candida* infection [92, 93], showed a high incidence (21.1% [26/123]) in this review. Contaminated foreign body caused direct insemination of* Candida* species into the uterus. Some authors suspect that cervical cerclage, another foreign body during pregnancy, is also a risk factor [6, 91]. However, the most prevalent predisposing condition was pPROM, which leads to an ascending infection of* Candida* species colonized in the vagina. The incidence of* Candida* species in the amniotic fluid of patients with pPROM was reportedly 5% using polymerase chain reaction and 3.2% using culture-based methods [93], which are higher than those of patients with preterm labor with intact membranes (1.2% and 0.6%, resp.) [94]. However, whether cervical cerclage and pPROM are risk factors is unclear because cervical incompetence, which is the indication for cervical cerclage, and pPROM could result from chorioamnionitis. Case reports of chorioamnionitis with* C. glabrata* associated with IVF have been increasing recently. A recent review reported that 65% of cases of* C. glabrata* chorioamnionitis were associated with IVF [74]. Our study showed that 20.3% (25/123) of cases with C*andida* chorioamnionitis were associated with IVF and that* C. glabrata*,* C. albicans*,* C. parapsilosis*,* C. kefyr*, and* C. famata* were identified. Several steps of the IVF procedure can contaminate the fertilized embryo or introduce the pathogen into the uterine cavity, although the risk of chorioamnionitis after IVF has not been well studied yet [95]. A case report showed that the eradication of* Candida* species in the vagina led to the birth of a healthy infant after stillbirth due to* Candida* chorioamnionitis [85]. The insemination of* Candida* species colonized in the skin through transabdominal amniocentesis could be another risk factor, as two authors suspected that the amniocentesis procedure itself led to the* Candida* chorioamnionitis [26, 57]. Amniocentesis provides an early diagnosis and an opportunity for aggressive management, including antenatal treatment, as described later.* Candida* chorioamnionitis may manifest as preterm labor, pPROM, or cervical incompetence without fever, while 39% (48/123) of cases in this study were antenatally diagnosed using amniotic fluid culture obtained through transabdominal amniocentesis. A recent case report also showed that the collection of amniotic fluid sludge succeeded to detect* C. albicans* [96]. Thus, we recommend performing transabdominal amniocentesis under strict aseptic conditions in cases of preterm labor or pPROM.

Since the information of* Candida* colonization in vagina was missing in most cases of the literature review, we were unable to evaluate the effect of* Candida* colonization in vagina on* Candida* chorioamnionitis in this study. A recent study, which showed recurrent vaginal colonization with* C. albicans* in early pregnancy was a risk factor for preterm delivery and low birth weight, indicates that the routine screening and consequent treatment for* Candida* colonization can be useful to improve pregnancy outcomes [96]. Although it is still unknown that vaginal colonization with* Candida* is associated with* Candia* chorioamnionitis, we also suggest that the screening and treatment for* Candida* colonization in vagina in early pregnancy is a reasonable strategy to prevent ascending infection.

### 4.3. Fetal/Neonatal Outcome

Almost 30% of singletons and 50% of twins born after 22 weeks died, even after the year 2000. Immaturity and severe fetal inflammation could be causes of the high mortality rate. As shown in Table 4, among the singletons delivered after 22 weeks of gestation, almost half were born before 28 weeks.

### 4.4. Antenatal Treatment

Antenatal treatment for* Candida* chorioamnionitis is challenging. Although the maternal administration of various antifungal agents via various routes has been conducted, only half of cases resulted in the delivery of live infants. Fluconazole was the most popular agent used in seven cases. However, fluconazole has less activity against* C. glabrata,* the second most prevalent species, and whether fluconazole crosses the placenta is unknown [97]. The use of high-dose systemic fluconazole during the first trimester has the potential risk of teratogenic effects on the fetus [97, 98], although a cohort study showed that low-dose fluconazole was not associated with birth defect [99]. Thus, amphotericin B is recommended as the first-line treatment in cases of invasive candidiasis in pregnant women and neonatal candidiasis [98]. The advantages of amphotericin B include that it crosses the placenta, has no reported adverse effects in humans [97], and is generally effective against* C. glabrata*. One case report showed that intravenous amphotericin B treatment resulted in pregnancy extension by 4 weeks and the delivery of live infants with no signs of inflammatory changes in the placenta or umbilical cord [89], although three other cases resulted in stillbirth [76, 87, 88]. Micafungin was used in one case, although its efficacy and safety in pregnant women are unclear, and whether it crosses the placenta remains unknown. However, its high molecular weight (approximately 1292 for the sodium salt), low lipid solubility, and very high protein binding ability should limit its transfer to the fetus [97]. Some cases of successful treatment with intra-amniotic administration were also reported [49, 71]. Transcervical intra-amniotic amphotericin B administration succeeded in extending a pregnancy by 2 weeks and resulted in the delivery of a healthy infant [49]. Transabdominal intra-amniotic fluconazole treatment with oral and vaginal administration successfully extended pregnancy by 6–8 weeks and resulted in the delivery of live infants [71]. Sheep studies showed that intra-amniotic fluconazole treatment prevented systemic inflammation and cerebral inflammation and injury [100, 101]. These reports indicate that intra-amniotic treatment is an antenatal treatment to be considered. We suggest amphotericin B should be chosen as a first-line agent and fluconazole should be avoided due to the possibility of adverse outcomes for the fetus [97].

## 5. Conclusion

*Candida* chorioamnionitis may manifest as preterm labor, pPROM, or cervical incompetence without fever. Therefore,* Candida* chorioamnionitis should be considered in pregnant women with these symptoms, even in the absence of fever, especially in those with early gestation with a retained IUCD, pregnancy after IVF, a history of amniocentesis, or cervical cerclage and preterm delivery. The preterm birth and fetal/neonatal mortality rates are high, and antenatal treatment has yet to be established.

## Figures and Tables

**Table 1:** Clinical features of nine cases of *Candida* chorioamnionitis in our hospital.

Case	Age (y)	GA at delivery	Species	Positive culture	Predisposing conditions	Clinical signs	BW (g)	CCC	Neonatal outcome
1	31	21 w 6 d	*Candida albicans*	Amniotic fluid	IVF	Preterm labor and afebrile WBC: 16,500/mm^3^ CRP: 0.51 mg/dL	490	No	Artificial abortion
2	31	22 w 6 d	*C. glabrata*	Amniotic fluid	IVF	Preterm labor and afebrile WBC: 11,600/mm^3^ CRP: 1.68 mg/dL	594	Yes	Death on day 59
3	28	23 w 0 d	*C. famata*	Amniotic fluid	Smoking	Preterm labor and afebrile WBC: 16,400/mm^3^ CRP: 0.68 mg/dL	498	No	Hydrocephalus after IVH
4	35	23 w 3 d	*C. albicans*	Amniotic fluid	IVF Gestational diabetes	Preterm labor and afebrile WBC: 11,700/mm^3^ CRP: 3.71 mg/dL	580	Yes	Neonatal death
5	28	23 w 4 d	*C. albicans*	Amniotic fluid	Pregestational diabetes	Preterm labor and afebrile WBC: 12,600/mm^3^ CRP: 3.8 mg/dL	536	No	Brain atrophy and CLD
6	33	25 w 2 d	*C. albicans*	Amniotic fluid	None	Preterm labor and afebrile WBC: 15,400/mm^3^ CRP: 7.51 mg/dL	786	No	PDA ligation
7	26	26 w 6 d	*C. albicans*	Amniotic fluid	None	Preterm labor and afebrile WBC: 9,800/mm^3^ CRP: 3.05 mg/dL	924	Yes	CLD
8	36	28 w 2 d	*C. albicans*	Umbilical blood	Di-Di twins	Preterm labor and afebrile WBC: 9,500/mm^3^ CRP: 3.34 mg/dL	1,290/1,168	No	Normal Only one twin had the infection
9	38	33 w 3 d	*C. glabrata*	Amniotic fluid	pPROM	Afebrile, WBC: 10,200/mm^3^ CRP: 2.68 mg/dL	1,912	No	Normal

GA, gestational age; BW, birth weight; CCC, congenital cutaneous candidiasis; IVF, in vitro fertilization; Di-Di twin, dichorionic diamniotic twin; WBC, white blood cell; CRP, C-reactive protein; IVH, intraventricular hemorrhage; CLD, chronic lung disease; PDA, patent ductus arteriosus; pPROM, preterm premature rupture of membranes.

**Table 2:** Species identified in the literature review excluding 22 unidentified cases (*n* = 101).

Species	*n* (%)
*Candida albicans*	72/101 (71.3)
*C. albicans alone*	70/101 (69.3)
Coinfection with *C. parapsilosis*	2/101 (2)
*C. glabrata*	22/101 (21.7)
*C. tropicalis*	3/101 (3)
*C. lusitaniae*	2/101 (2)
*C. famata*	1/101 (1)
*C. kefyr*	1/101 (1)

**Table 3:** Maternal clinical features in the literature review.

	*n* (%)
Maternal age, years; median (range)	29 (16–47)
Singleton/twins	102/21
Predisposing condition	
pPROM	31/123 (25.2)
IUCD	26/123 (21.1)
IVF	25/123 (20.3)
History of amniocentesis during current pregnancy	11/123 (8.9)
Cervical cerclage	8/123 (6.5)
Pregestational or gestational diabetes	5/123 (4.1)
History of treatment for vaginal candidiasis during current pregnancy	14/123 (11.4)
Symptoms	
Preterm labor with intact membranes	52/123 (42.3)
Fever	16/123 (13)
Cervical dilatation	11/123 (8.9)
Abdominal pain	7/123 (5.7)
Vaginal bleeding	6/123 (4.9)
Reduced fetal movement	2/123 (1.6)
None	12/123 (9.8)

pPROM, preterm premature rupture of membranes; IUCD, intrauterine contraceptive device; IVF, in vitro fertilization.

**Table 4:** Gestational age at delivery in the literature review.

Gestational age (weeks)	Singletons (*n* = 100)	Twins (*n* = 21)
<22	27/100 (27%)	5/21 (23.8%)
22–36	60/100 (60%)	16/21 (76.2%)
22-23	11/100 (11%)	2/21 (9.5%)
24–27	23/100 (23%)	4/21 (19%)
28–31	13/100 (13%)	7/21 (33.3%)
32–36	13/100 (13%)	3/21 (14.3%)
≥37	13/100 (13%)	0/21 (0%)

2 singletons with unknown gestational age were excluded.

**Table 5:** Antenatal treatment for *Candida* chorioamnionitis.

GA at diagnosis (weeks)	GA at delivery (weeks)	Species	Antifungal agent	Administration route	Fetal/neonatal outcomes	Reference
19	19	*Candida albicans*	Fluconazole	Intravenous	Stillbirth	[74]
19	21	*C. albicans*	Fluconazole	Oral	Stillbirth	[75]
21	22	*C. albicans*	Fluconazole	Oral	Stillbirth	[64]
21	27	*C. albicans*	Fluconazole	Transabdominal intra-amniotic, oral, and vaginal	Alive	[71]
23	31	*C. albicans*	Fluconazole	Transabdominal intra-amniotic, oral, and vaginal	Alive	[71]
26	29	*C. glabrata*	Fluconazole	Intravenous	Alive (PVL)/alive	[81]
18	23	*C. glabrata*	Amphotericin B	Intravenous	Stillbirth	[88]
21	24	*C. glabrata*	Amphotericin B	Intravenous	Stillbirth/stillbirth	[87]
24	24	*C. glabrata*	Amphotericin B	Intravenous	Died on day 42	[76]
24	28	*C. glabrata*	Amphotericin B	Intravenous	Alive/alive	[89]
27	29	*C. albicans*	Amphotericin B	Transcervical intra-amniotic	Alive	[44]
21	21	*C. albicans*	Ketoconazole	Not described	Stillbirth	[91]
26	28	*C. glabrata*	Micafungin	Intravenous	Alive	[41]

GA, gestational age; PVL, periventricular leukomalacia.

## References

[1] Kiss H., Petricevic L., Husslein P. (2004). Prospective randomised controlled trial of an infection screening programme to reduce the rate of preterm delivery. *British Medical Journal*.

[2] Roberts C. L., Rickard K., Kotsiou G., Morris J. M. (2011). Treatment of asymptomatic vaginal candidiasis in pregnancy to prevent preterm birth: an open-label pilot randomized controlled trial. *BMC Pregnancy and Childbirth*.

[3] Barton M., Shen A., OBrien K., Robinson JL., Davies HD., Simpson K. (2017). Early onset invasive candidiasis in extremely low birth weight infants: perinatal acquisition predicts poor outcome. *Clin Infect Dis*.

[4] Benirschke K., Raphael S. I. (1958). Candida albicans infection of the amniotic sac. *American Journal of Obstetrics & Gynecology*.

[5] Qureshi F., Jacques S. M., Bendon R. W. (1998). Candida funisitis: a clinicopathologic study of 32 cases. *Pediatric and Developmental Pathology*.

[6] Whyte R. K., Hussain Z., DeSa D. (1982). Antenatal infections with Candida species. *Archives of Disease in Childhood*.

[7] Maki Y., Furukawa S., Kodama Y., Sameshima H., Ikenoue T. (2015). Amniocentesis for threatened preterm labor with intact membranes and the impact on adverse outcome in infants born at 22 to 28 weeks of gestation. *Early Human Development*.

[8] Belter L. F. (1959). Thrush of the umbilical cord. *Obstet Gynecol*.

[9] Galton M., Benirschke K. (1960). The implication of candida albicans infection of the amniotic sac. *BJOG: An International Journal of Obstetrics & Gynaecology*.

[10] Dvorak A. M., Gavaller B. (1966). Congenital systemic candidiasis, report of a case. *The New England Journal of Medicine*.

[11] Abaci F., Aterman K. (1966). Monilial infection of the umbilical cord. *Obstetrics & Gynecology*.

[12] Albarracin N. S., Patterson W. S., Haust M. D. (1967). Candida albicans infection of the placenta and fetus, Report of a case. *Obstetrics & Gynecology*.

[13] Schweid A. I., Hopkins B. G. (1968). Monilial chorionitis associated with an intrauterine contraceptive device. *Obstetrics & Gynecology*.

[14] Rhatigan R. M. (1968). Congenital cutaneous candidiasis. *American Journal of Diseases of Children*.

[15] Lopez E., Aterman K. (1968). Intra-Uterine Infection by Candida. *American Journal of Diseases of Children*.

[16] Ho C.-Y., Aterman K. (1970). Infection of the fetus by Candida in a spontaneous abortion. *American Journal of Obstetrics & Gynecology*.

[17] Misenhimer H. R., Garcia-Bunuel R. (1969). Failure of intrauterine contraceptive device and fungal infection in the fetus. *Obstetrics & Gynecology*.

[18] Schirar A., Rendu C., Vielh J. P., Gautray J. P. (1974). Congenital mycosis (Candida albicans). *Neonatology*.

[19] Brandsma M. A. C., Braaksma J. T., van der Harten J. J. (1975). Immature delivery after intrauterine Candida albicans infection. *European Journal of Obstetrics & Gynecology and Reproductive Biology*.

[20] Rudolph N., Tariq A. A., Reale M. R., Goldberg P. K., Kozinn P. J. (1977). Congenital cutaneous candidiasis. *JAMA Dermatology*.

[21] Quirke P., Hwang W. S., Validen G. C. (1980). Congenital Torulopsis glabrata infection in man. *American Journal of Clinical Pathology*.

[22] Buchanan R., Sworn M. J., Noble A. D. (1979). Abortion associated with intrauterine infection by candida albicans case report. *BJOG: An International Journal of Obstetrics & Gynaecology*.

[23] Johnson D. E., Thompson T. R., Ferrieri P. (1981). Congenital Candidiasis. *American Journal of Diseases of Children*.

[24] Nagata K., Nakamura Y., Hosokawa Y. (1981). Intrauterine candida infection in premature baby. *Acta Pathologica Japonica*.

[25] Delprado W. J., Baird P. J., Russell P. (1982). Placental candidiasis: Report of three cases with a review of the literature. *Pathology*.

[26] Delaplane D., Wiringa K. S., Shulman S. T., Yogev R. (1983). Congenital mucocutaneous candidiasis following diagnostic amniocentesis. *American Journal of Obstetrics & Gynecology*.

[27] Bittencourt A. L., dos Santos W. L. C., de Oliveira C. H. (1984). Placental and fetal candidiasis - Presentation of a case of an abortus. *Mycopathologia*.

[28] Honoré L. H. (1984). Placental candidiasis: Report of two cases, one associated with an IUCD in situ. *Contraception*.

[29] Romero R., Reece E. A., Duff G. W., Coultrip L., Hobbins J. C. (1985). Prenatal diagnosis of Candida albicans chorioamnionitis. *American Journal of Perinatology*.

[30] Mamlok R. J., Joan Richardson C., Mamlok V., Nichols M. M., Goldblum R. M. (1985). A case of intrauterine pulmonary candidiasis. *Pediatric Infectious Disease*.

[31] Spaun E., Klünder K. (1986). Candida Chorioamnionitis and Intra‐Uterine Contraceptive Device. *Acta Obstetricia et Gynecologica Scandinavica*.

[32] Bruner J. P., Elliott J. P., Kilbride H. W., Garite T. J., Knox G. E. (1986). Candida chorioamnionitis diagnosed by amniocentesis with subsequent fetal infection. *American Journal of Perinatology*.

[33] Faix R. G., Naglie R. A., Barr M. (1986). Intrapleural inoculation of candida in an infant with congenital cutaneous candidiasis. *American Journal of Perinatology*.

[34] Smith C. V., Horenstein J., Platt L. D. (1988). Intraamniotic infection with Candida albicans associated with a retained intrauterine contraceptive device: A case report. *American Journal of Obstetrics & Gynecology*.

[35] Bider D., Ben-Rafael Z., Barkai G., Mashiach S. (1989). Intrauterine fetal death apparently due to Candida chorioamnionitis. *Archives of Gynecology and Obstetrics*.

[36] Morgan M. A., Pippitt C. H., Thurnau G. R. (1989). Antenatal diagnosis of candida chorioamnionitis. *Southern Medical Journal*.

[37] Donders G. G. G., Moerman P., Caudron J., Van Assche F. A. (1991). Intra-uterine Candida infection: a report of four infected fetusses from two mothers. *European Journal of Obstetrics & Gynecology and Reproductive Biology*.

[38] Schwartz D. A., Reef S. (1990). Candida albicans placentitis and funisitis: Early diagnosis of congenital candidemia by histopathologic examination of umbilical cord vessels. *The Pediatric Infectious Disease Journal*.

[39] Arbegast K. D., Lamberty L. F., Koh J. K., Pergram J. M., Braddock S. W. (1990). Congenital candidiasis limited to the nail plates. *Pediatric Dermatology*.

[40] Mazor M., Chaim W., Pak I., Goldstein D. (1992). Intraamniotic infection with Candida albicans associated with a retained intrauterine device: a case report. *Obstetrics, Gynaecology and Reproductive Medicine*.

[41] Chaim W., Mazor M., Meril T., Peleg R., Maor E. (1993). Late miscarriage and intraamniotic candidiasis in a woman with a retained intrauterine contraceptive device. *Archives of Gynecology and Obstetrics*.

[42] Mazor M., Chaim W., Shinwell E., Glezerman M. (1993). Asymptomatic amniotic fluid invasion with Candida albicans in preterm premature rupture of membranes: Implications for obstetric and neonatal management. *Acta Obstetricia et Gynecologica Scandinavica*.

[43] Ng P. C., Siu Y. K., Lewindon P. J., Wong W., Cheung K. L., Dawkins R. (1994). Congenital Candida pneumonia in a preterm infant. *Journal of Paediatrics and Child Health*.

[44] Shalev E., Battino S., Romano S., Blondhaim O., Ben-Ami M. (1994). Intraamniotic infection with Candida albicans successfully treated with transcervical amnioinfusion of amphotericin. *American Journal of Obstetrics & Gynecology*.

[45] Van Winter J. T., Ney J. A., Ogburn P. L., Johonson R. V. (1994). Preterm labor and congenital candidiasis, A case report. *J Reprod Med*.

[46] Jin Y., Endo A., Shimada M. (1995). Congenital systemic candidiasis. *The Pediatric Infectious Disease Journal*.

[47] Nichols A., Khong T. Y., Crowther C. A. (1995). Candida tropicalis chorioamnionitis. *American Journal of Obstetrics & Gynecology*.

[48] Raval D. S., Barton L. L., Hansen R. C., Kling P. J. (1995). Congenital cutaneous candidiasis: Case report and review. *Pediatric Dermatology*.

[49] Khambadkone S. M., Dixit K. M., Divekar A., Joshi S. M., Irani S. F., Desai M. (1996). Congenital candidiasis. *Indian Pediatrics*.

[50] DiLorenzo D. J., Wong G., Ludmir J. (1997). Candida lusitaniae chorioamnionitis in a bone marrow transplant patient. *Obstetrics & Gynecology*.

[51] Engelhart C. M., Van De Vijver N. M. A., Nienhuis S. J., Hasaart T. H. M. (1998). Fetal Candida sepsis at midgestation: A case report. *European Journal of Obstetrics & Gynecology and Reproductive Biology*.

[52] Berry D. L., Olson G. L., Wen T. S., Belfort M. A., Moise K.J. J. (1997). Candida chorioamnionitis: A report of two cases. *The Journal of Maternal-Fetal Medicine*.

[53] Rivasi F., Gasser B., Bagni A., Ficarra G., Negro R. M., Philippe E. (1998). Placental candidiasis: Report of four cases, one with villitis. *APMIS-Acta Pathologica, Microbiologica et Immunologica Scandinavica*.

[54] Pradeepkumar V. K., Rajadurai V. S., Tan K. W. (1998). Congenital Candidiasis: Varied Presentations. *Journal of Perinatology*.

[55] Roqué H., Abdelhak Y., Young B. K. (1999). Intra amniotic candidiasis. Case report and meta-analysis of 54 cases. *Journal of Perinatal Medicine*.

[56] Waguespack-LaBiche J., Chen S.-H., Yen A. (1999). Disseminated congenital Candidiasis in a premature infant. *JAMA Dermatology*.

[57] Rode M. E., Morgan M. A., Ruchelli E., Forouzan I. (2000). Candida chorioamnipnitis after serial therapeutic amniocenteses: a possible association. *Journal of Perinatology*.

[58] Horn L.-C., Nenoff P., Ziegert M., Höckel M. (2001). Missed abortion complicated by Candida infection in a woman with rested IUD. *Archives of Gynecology and Obstetrics*.

[59] Segal D., Gohar J., Huleihel M., Mazor M. (2001). Fetal death associated with asymptomatic intrauterine Candida albicans infection and a retained intrauterine contraceptive device. *Infectious Diseases*.

[60] Barth T., Broscheit J., Bussen S., Dietl J. (2002). Maternal sepsis and intrauterine fetal death resulting from Candida tropicalis chorioamnionitis in a woman with a retained intrauterine contraceptive device. *Acta Obstetricia et Gynecologica Scandinavica*.

[61] Diana A., Epiney M., Ecoffey M., Pfister R. E. (2004). "White dots on the placenta and red dots on the baby": Congential cutaneous candidiasis - A rare disease of the neonate. *Acta Paediatrica*.

[62] Matsuzawa S., Ohyama M., Kawataki M. (2005). Congenital candida clabrata infection without specific nodules on the placenta and umbilical cord. *The Pediatric Infectious Disease Journal*.

[63] Freydiere A. M., Piens M. A., Andre J. M., Putet G., Picot S. (2005). Successful treatment of Candida glabrata peritonitis with fluconazole plus flucytosine in a premature infant following in vitro fertilization. *European Journal of Clinical Microbiology & Infectious Diseases*.

[64] Crawford J. T., Pereira L., Buckmaster J., Gravett M. G., Tolosa J. E. (2006). Amniocentesis results and novel proteomic analysis in a case of occult candidal chorioamnionitis. *The Journal of Maternal-Fetal and Neonatal Medicine*.

[65] Meizoso T., Rivera T., Fernández-Aceñero M. J., Mestre M. J., Garrido M., Garaulet C. (2008). Intrauterine candidiasis: Report of four cases. *Archives of Gynecology and Obstetrics*.

[66] Lee H. S. J., Lo A. W. I. (2011). Placental and fetal candidiasis associated with intrauterine contraceptive device in situ. *Pathology*.

[67] Canpolat F. E., Çekmez F., Tezer H. (2011). Chorioamnionitis and neonatal sepsis due to Candida tropicalis. *Archives of Gynecology and Obstetrics*.

[68] Huang M., Cham E. M., Eppes C. S., Gerber S. E., Reed K. D., Ernst L. M. (2012). Placental and fetal findings in intrauterine Candida lusitaniae infection following in vitro fertilization and embryo transfer. *Pediatric and Developmental Pathology*.

[69] Özer E., Ünlü M., Erşen A., Gülekli B. (2013). Intrauterine fetal loss associated with Candida glabrata chorioamnionitis: Report of two cases. *Turk Patoloji Dergisi/Turkish Journal of Pathology*.

[70] Ito F., Okubo T., Yasuo T. (2013). Premature delivery due to intrauterine Candida infection that caused neonatal congenital cutaneous candidiasis: A case report. *Journal of Obstetrics and Gynaecology Research*.

[71] Bean L. M., Jackson J. R., Dobak W. J., Beiswenger T. R., Thorp J. A. (2013). Intra-amniotic fluconazole therapy for Candida albicans intra-amniotic infection. *Obstetrics & Gynecology*.

[72] Iwatani S., Murakami Y., Mizobuchi M. (2014). Successful management of an extremely premature infant with congenital candidiasis. *American Journal of Perinatology Reports*.

[73] Alfei A., Rizzo A., Cavanna C., Lallitto F., Spinillo A. (2014). Candida glabrata and pre-term premature rupture of membrane complicating in vitro pregnancy: case report and confirmation of mother to neonate transmission. *Archives of Gynecology and Obstetrics*.

[74] Ganer Herman H., Mevorach Zussman N., Krajden Haratz K., Bar J., Sagiv R. (2015). Candida glabrata chorioamnionitis following in vitro fertilization: Review of the literature. *Gynecologic and Obstetric Investigation*.

[75] Poliquin V., Al-Sulmi E., Menticoglou S. (2015). Intra-amniotic infection involving Candida albicans subsequent to emergency cerclage: a case series. *Canadian Journal of Infectious Diseases & Medical Microbiology*.

[76] Garcia-Flores J., Cruceyra M., Cañamares M., Garicano A., Nieto O., Tamarit I. (2016). Candida chorioamnionitis: Report of two cases and review of literature. *Journal of Obstetrics & Gynaecology*.

[77] Levin S., Zaidel L., Bernstein D. (1978). Intrauterine infection of fetal brain by candida. *American Journal of Obstetrics & Gynecology*.

[78] Sfameni S. F., Talbot J. M., Chow S. L., Brenton L. A., Scurry J. P. (1997). Candida G lab rata chorioamnionitis following in vitro fertilization and embryo transfer. *ANZJOG*.

[79] Donders G. G. G., Gordts S., Cornelis A., Moerman P. (1997). Intrauterine candidiasis in a twin pregnancy after myomectomy, in vitro fertilization and embryo transfer. *Archives of Gynecology and Obstetrics*.

[80] Friebe-Hoffmann U., Bender D. P., Sims C. J., Rauk P. N. (2000). Candida albicans chorioamnionitis associated with preterm labor and sudden intrauterine demise of one twin: a case report. *Obstetrics, Gynaecology and Reproductive Medicine*.

[81] Arai H., Goto R., Matsuda T. (2002). Case of congenital infection with Candida glabrata in one infant in a set of twins. *Pediatrics International*.

[82] Ibara A. S., Marcorelles P., Le Martelot M. T. (2004). Two cases of systemic candida glabrata infection following in vitro fertilization and embryo transfer. *European Journal of Clinical Microbiology & Infectious Diseases*.

[83] Krallis N., Tzioras S., Giapros V. (2006). Congenital candidiasis caused by different candida species in a dizygotic pregnancy. *The Pediatric Infectious Disease Journal*.

[84] Carmo K. B., Evans N., Isaacs D. (2007). Congenital candidiasis presenting as septic shock without rash. *Archives of Disease in Childhood*.

[85] Asemota O. A., Nyirjesy P., Fox R., Sobel J. D. (2011). Candida glabrata complicating in vitro pregnancy: Successful management of subsequent pregnancy. *Fertility and Sterility*.

[86] Pineda C., Kaushik A., Kest H., Wickes B., Zauk A. (2012). Maternal sepsis, chorioamnionitis, and congenital Candida kefyr infection in premature twins. *The Pediatric Infectious Disease Journal*.

[87] Jackel D., Lai K. (2013). Candida glabrata sepsis associated with chorioamnionitis in an in vitro fertilization pregnancy: Case report and review. *Clinical Infectious Diseases*.

[88] Akhanoba F., MacDougall J., Mathur R., Hassan W. (2014). Severe systemic candidiasis following immunomodulation therapy in in vitro fertilisation-embryo transfer (IVF-ET). *BMJ Case Reports*.

[89] Tan S. Q., Ng O. T., Khong C. C. (2015). Candida glabrata sepsis associated with chorioamnionitis in an IVF twin pregnancy: should we deliver?. *Journal of Obstetrics and Gynaecology Research*.

[90] Chen W., Chen S., Tsai S., Tsao P., Tang R., Soong W. (2015). Congenital systemic fungus infection in twin prematurity—a case report and literature review. *American Journal of Perinatology Reports*.

[91] Chaim W., Mazor M., Wiznitzer A. (1992). The prevalence and clinical significance of intraamniotic infection with Candida species in women with preterm labor. *Archives of Gynecology and Obstetrics*.

[92] Kim S. K., Romero R., Kusanovic J. P. (2010). The prognosis of pregnancy conceived despite the presence of an intrauterine device (IUD). *Journal of Perinatal Medicine*.

[93] DiGiulio D. B., Romero R., Kusanovic J. P. (2010). Prevalence and diversity of microbes in the amniotic fluid, the fetal inflammatory response, and pregnancy outcome in women with preterm pre-labor rupture of membranes. *American Journal of Reproductive Immunology*.

[94] DiGiulio D. B., Romero R., Amogan H. P. (2008). Microbial prevalence, diversity and abundance in amniotic fluid during preterm labor: a molecular and culture-based investigation. *PLoS ONE*.

[95] McDonald S. D., Murphy K., Beyene J., Ohlsson A. (2005). Perinatel outcomes of singleton pregnancies achieved by in vitro fertilization: a systematic review and meta-analysis. *Journal of Obstetrics and Gynaecology Canada*.

[96] Farr A., Kiss H., Holzer I., Husslein P., Hagmann M., Petricevic L. (2015). Effect of asymptomatic vaginal colonization with Candida albicans on pregnancy outcome. *Acta Obstetricia et Gynecologica Scandinavica*.

[97] Briggs G. G., Freeman R. K. (2015). *Drug in pregnancy and lactation*.

[98] Pappas P. G., Kauffman C. A., Andes D. R. (2016). Clinical practice guideline for the management of candidiasis: 2016 update by the infectious diseases society of America. *Clinical Infectious Diseases*.

[99] Mølgaard-Nielsen D., Pasternak B., Hviid A. (2013). Use of oral fluconazole during pregnancy and the risk of birth defects. *The New England Journal of Medicine*.

[100] Ophelders D. R. M. G., Gussenhoven R., Lammens M. (2016). Neuroinflammation and structural injury of the fetal ovine brain following intra-amniotic Candida albicans exposure. *Journal of Neuroinflammation*.

[101] Maneenil G., Payne M. S., Senthamarai Kannan P. (2015). Fluconazole treatment of intrauterine Candida albicans infection in fetal sheep. *Pediatric Research*.

